# Characteristics, risk management and GMP standards of pharmaceutical companies in China

**DOI:** 10.3389/fpubh.2023.1103555

**Published:** 2023-03-08

**Authors:** Hong Chen, Lijian Qin, Cong Jiang, Mingshuai Qin, Yanming Sun, Jingjing Luo

**Affiliations:** ^1^Anhui University of Finance and Economics, Bengbu, Anhui, China; ^2^University of Waterloo, Waterloo, ON, Canada; ^3^Faculty of Economics, VSB-Technical University of Ostrava, Ostrava, Czechia; ^4^Institute for Global Innovation and Development/School of Urban and Regional Science/Institute of Eco-Chongming, East China Normal University, Shanghai, China; ^5^Anhui Center for Drug Evaluation and Inspection, Hefei, China

**Keywords:** pharmaceutical company characteristics, risk management, GMP, pharmaceutical company supervision, China

## Abstract

The Good Manufacturing Practice (GMP) is one of the gold standards by which governments worldwide judge modern pharmaceutical companies' production processes and product-safety standards. However, in all the nations, it is difficult to obtain real data about GMP inspection results, so conducting the related research is impossible. Taking advantage of a rare chance to obtain the on-site GMP inspection results in China, we have been able to initiate an empirical analysis of how company characteristics and risk management affect the GMP inspection results of certain pharmaceutical companies. The 2SLS method regression was employed in this study. Our four main findings are as follows. First, compared with Chinese state-owned companies, foreign commercial and private enterprises are held to higher standards. Second, the GMP inspection results tend to be better for those enterprises whose main sources of capital are not dependent on bank loans. Third, enterprises with higher fixed assets tend to receive the better GMP inspection results. Fourth, the longer the quality authorized staff has worked in a company, the better the GMP inspection results expected of that enterprise. These findings offer insights into inspections and production improvements in China and other GMP-compliant countries.

## 1. Introduction

Drug safety concerns have repeatedly emerged in China in recent years (1, 2). The drug production process is the source of drug quality and safety, and strengthening the supervision of pharmaceutical companies' production processes is the basis for ensuring drug safety (3). Good Manufacturing Practice (GMP) form the basis for a mandatory management system that most pharmaceutical companies around the world have agreed to follow to ensure their drugs' quality and consumer confidence. However, how can the GMP supervision of pharmaceutical companies be improved to ensure the pertinence of supervision? There is no empirical research on this issue globally because the confidentiality and non-publication of GMP inspection results make it difficult for the public. If a company could find out the characteristics that affect its GMP results, then supervising its manufacturing processes would become much more focused and relevant to meeting the GMP standards in future inspections. An unprecedented chance to obtain actual inspection results for some of China's GMP companies has given us a rare opportunity to carry out empirical testing of how these companies' external characteristics affect their compliance with GMP. This work is of great significance for improving the efficiency of GMP supervision, ensuring drug quality, and improving drug regulatory policies.

From a company's point of view, the whole process of government drug supervision, from the company's application for marketing to patient use, can be divided into three stages: product registration and approval, enterprise production, and circulation and sales (4–6). Based on data availability, most relevant research focuses on either the first stage (registration and approval) or the third stage (circulation and sales). The drug supervision research in the first stage focuses on the factors that influence the length of time after the initial application for a new drug to reach market, which is also a concern for pharmaceutical companies. As China's drug research and development capabilities are relatively weak, and there are fewer original drugs, the research at this stage mainly comes from developed countries such as the United States and Japan (7–10). Liu and Wang (11), Kelly et al. (12) and Wada et al. (13) researched how to strengthen the supply chain supervision of drug circulation in the third stage (marketing and sales).

Globally, the scant documentation available for researching the processes of pharmaceutical companies in the second stage (i.e., drug production) is based on work notes and papers from the Center for Drug Evaluation written by the China National Medical Products Administration inspectors. All GMP inspectors are government employees, but only in China their promotions are linked to publishing papers on their work. Luo et al. (14), Hu et al. (15) and Wang et al. (16), based on inspections of 46 and 197 pharmaceutical companies in Anhui Province and Liaoning Province of China, respectively, found that the maintenance of production equipment and laboratory management was insufficient, which led to the relatively standardized GMP of pharmaceutical manufacturers. The cost of manually cleaning production equipment is lower, and that of automatic cleaning is higher. Liu et al. (17), and Li and Fu (18) used the inspection results of 20 and 43 drug manufacturers in Shanghai, respectively, and found that most companies use manual cleaning methods, which have a high error rate. Although automatic cleaning works better, it is used only in big companies. Some enterprises even release some substandard materials for production. These production defects caused by human factors carry hidden dangers for drug quality and safety.

When Liu et al. (19), Lu et al. (20), and Cao and Wu (21) studied the on-site inspections of 26 enterprises in the Qinghai Province and 34 enterprises in the Guangxi Zhuang Autonomous Region, China, respectively, they found that some the companies had insufficient staff training, factories and production facilities were laid out in inefficient configurations, missing risk reviews and assessments, poor attention to the storage and maintenance of items, and incomplete or sloppy the self-inspection measures and records needed for GMP records. Yang et al. (22) found that China's pharmaceutical industry has low barriers to entry, and 98% of pharmaceutical companies are small and medium-sized enterprises. Entering and exiting the industry are relatively frequent, and thus the consistency and high standards needed for product quality cannot be guaranteed, which increases the difficulty of drug quality management. It is necessary for China to establish a unified and authoritative modern food and drug regulatory system to improve the government's drug regulatory capabilities (23–25). Li and Liang (26) further found that companies must comply with GMP guidelines for production and set higher production standards to ensure drug quality.

With regard to government regulation, in a narrow sense, it is the management and restriction of economic behaviors (27–29). It is the behavior of social public institutions to supervise and restrict the activities of enterprises according to certain rules. Government regulations can be divided into social regulations and economic regulations. The social regulation is to ensure the safety of the production process, while the economic regulation is to regulate prices and services in order to promote market equity. In a broad sense, government regulation is generally interpreted as the incentive and constraint of all public rights on private rights (30, 31). It is undeniable that when there are market failures which are hard to be effectively corrected, government regulation is usually a reasonable choice. This also serves as an important basis for explaining the necessity of government regulation. In addition, government regulation is considered to be established by politicians and regulators to pursue their own interests (32, 33). The cumbersome approval procedures, strict access barriers, and various regulation fees give regulators considerable discretion, allowing them to use these powers for “rent-seeking”. Further, regulators typically have limited resources for enforcement. Literature to date has shown that even with those limitations a wide variety of instruments—including inspections, warnings, penalties, and sanctions—can be effective in inducing compliance (33, 34).

The existing literature has laid a good foundation for this present study, but three problems need to be solved urgently: (1) the lack of empirical research based on the results of GMP inspection results; and (2) the lack of studies focused on the supervision of the drug production process, that is, the second stage; (3) the sample size is small in the only few Chinese papers. The main obstacle to solving these problems is that it is challenging to obtain GMP inspection results, for some reasons. First, the inspection results are not made public. For example, the US FDA stipulates a 10-year confidentiality period for inspection results, but even after it, GMP inspectors cannot expose any details that would let the outside world know which pharmaceutical company is involved. Second, inspectors' qualifications are high; thus, the requirements of inspectors are also relatively strict. After receiving an excellent education and passing the qualification examination, inspectors are then qualified for GMP inspection. Therefore, compared with the responsibilities of typical social-surveys investigators, the responsibilities of GMP inspectors are very high. Third, the potential cost of an investigation is vast. For instance, sometimes, even if the investigator wears sterile protective clothing, the inspected company may worry that the production environment could be contaminated. To eliminate potential risks due to an inspection, most companies need to pay a lot to maintain their production environment. Thus, the inspection process causes substantial extra costs, normally at least hundreds of thousands or even a million USD.

Based on the actual GMP inspection results of pharmaceutical companies in Anhui Province of China by the National Medical Products Administration, we the first conducted an empirical study on the second stage, that is, drug GMP supervision on a global scale, striving to solve the above problems to make exploratory contributions in the field. Our research goal is to empirically test whether a pharmaceutical company's characteristics and risk management impact its GMP inspections results. We will examine the three hypotheses. Hypothesis 1, compared with state-owned enterprises, foreign-owned and private enterprises have a higher degree of GMP regulation. Hypothesis 2, enterprises with a higher gross fixed asset value have a higher degree of GMP regulation. Hypothesis 3, the degree of GMP regulation is higher for enterprises whose main source of funding does not rely on bank loans. This study discusses the differences in the actual implementation effects of GMP regulations in pharmaceutical companies and explores the reasons for these differences. The findings in this paper are consistent with those of Liu et al. (17), Li and Fu (18), Liu et al. (19), and Lu et al. (20). However, these articles did not examine the company's characteristics and did not compare the GMP examination results with those of foreign and private companies. In addition, they did not examine the differences among enterprises of different sizes. We examine these aspects, which is an important contribution of this paper. These findings may reflect the regulation's actual shortcomings, so this research provides policy support and reference for improving the regulation. In addition, the empirical research in this article makes a foundational contribution to academic research or policy maker. The theoretical contribution of this paper is that it applies external signals theory to the regulation of quality in the production process of pharmaceutical companies, using external characteristics of companies as an entry point for regulation and improving the feasibility and effectiveness of government regulation. The findings of this paper have important implications not only for China but also for countries around the world, especially for developing countries in general. The rest of the article is arranged as follows: the second part introduces the development process of GMP; the third part is the theoretical framework and econometrics model; the fourth part is the data source and descriptive statistical analysis; the fifth part is the empirical results, and the sixth part is the brief conclusion and recommendations.

## 2. The development history of GMP

In formulating and improving GMP, the United States is in a leading position globally. The American public's awareness of the need for government oversight of consumable products began in 1906, when the American journalist and famous social reformer Upton Sinclair exposed the dirty inside story of Chicago's meat-processing industry. The slaughtering and processing environment was extremely unhygienic, and a large number of pork products made of diseased pigs were sold, some partially deteriorated, and some even containing mice (35). The report's original intention was to arouse everyone's attention to the harsh working environment and workers' poor conditions, and it immediately had a significant impact on all sectors of American society. Simultaneously, syrups and adult health products used for the treatment of infant colic had also been found to contain addictive ingredients such as ethanol or morphine. The U.S. Congress enacted the Pure Food and Drug Act that year, making the sale of adulterated (contaminated) meat or food an illegal act for the first time. This was the first time that products were regulated. For one thing, a label now had to be accurate, and any dangerous ingredients had to be marked. If labeling was inaccurate or false, its producers were liable to charges of misleading advertising. This bill directly led to establishing a new government agency in the United States—the Food and Drug Administration (FDA), which still has the power to seize illegal foods and drugs.

In 1935, sulfa drugs came out, and many manufacturers began to produce this new type of anti-infective treatment. Among patients who used the drug, 107 lost their lives within a year, most of them children. After inspection, the reason was found to be that a manufacturer had used the toxic solvent diethylene glycol in the drug's production (36). Congress reacted quickly and promulgated the Federal Food, Drug, and Cosmetic Act in 1938. For the first time, manufacturers had to certify products' safety before they were put on the market. In 1941, a U.S. pharmaceutical company produced sulfathiazole tablets that were contaminated with phenobarbital, a sedative and sleeping agent, which directly caused 300 deaths and injuries. This incident prompted the FDA to make a thorough revision of drug-production quality, which formed an embryonic form of GMP (37). During World War II, the amount of insulin and penicillin increased dramatically. To ensure the quality of such medicines, the United States required the FDA to test both before their release. Later, this rule was expanded to include all antibiotics requiring FDA certification. In the 1960s, the drug Thalidomide caused more than 10,000 cases of severe deformities in newborn babies, shocking the world (36). In response, in 1963, the US FDA promulgated the world's first “Good Manufacturing Practices for Drugs”, that is GMP, to strengthen drug safety. This event was a milestone in the history of the pharmaceutical industry, marking the point where it began to realize total quality management and laying a solid foundation for ensuring medicines' quality.

The core of quality control lies in people, not only managers but also ordinary employees. This point is illustrated by a case from 1992, when a generic drug scandal broke out in the United States. The illegal activities cited included destroying samples, falsifying production records, and concealing changes in production processes, and not only were company executives charged; regular employees were also subject to the resulting legal sanctions. In line with FDA policy, all guilty personnel were removed from the company and barred from future work in the pharmaceutical industry; moreover, all offenders were named, and their illegal acts were published on the FDA website. Now, when the company submits a listing application to the FDA, it must ensure in writing that no removed personnel participate in the project (38). In the same period, another company in the United States illegally produced cardiac catheters, resulting in the death of at least one patient and causing at least 20 patients to undergo emergency cardiac surgery. The court-imposed sentences of between 18 months and 2 years on the company's three executives, with the judge emphasizing that companies do not commit crimes, only people do. The judge also encouraged the managers in other companies who might be involved in similar illegalities to think twice, and desist (39). In reinforcing consistent high standards of behavior, GMP regard people's subjective initiative as the basis of standardized production, thereby strengthening the standardized operations of all managers and ordinary employees to ensure the quality of medicines.

In the several years of practice after the US GMP guidelines were first promulgated, the practice proved GMP's effeteness. Their intense vitality quickly emerged, and countries all over the world followed suit. The World Health Organization included this system in the appendix of the International Pharmacopeia in 1967, and promulgated its own GMP regulations in 1969, recommending that member states use them as a regulatory system for drug production. Later, in 1979, GMP were once again introduced to the World Health Assembly member states; they were also confirmed as a World Health Organization body of regulations. Australia in 1968, the United Kingdom in 1971, the European Union in 1972, and Japan in 1974 issued their own GMP regulations. So far, more than 100 countries and regions around the world have implemented GMP. After many revisions, the US GMP standard is currently the world's most detailed version, with complete indicators and the highest standards of implementation. The United States requires that all pharmaceutical manufacturers in the United States and pharmaceutical companies exporting to the United States comply with United States GMP standards.

China put forward its own GMP standards 20 years later than the United States. Keeping in mind the GMP of more-advanced countries, China's National Pharmaceutical Industry Corporation formulated China's first domestic GMP in 1982, then began to carry out GMP pilot work in its sub pharmaceutical companies. In the early days of the founding of the People's Republic of China, due to the planned economy era, China's pharmaceutical industry was relatively backward, and basic supply needs could not be met, so the quality concept was weak. With the deepening of reforms and opening up and learning from foreign experience, the Chinese government officially formulated GMP in 1988 as a product specification for medicines. It was revised in 1992 and 1998. In the same period, the China National Medical Products Administration was established in 1998, as an agency directly under the State Council and specializing in drug supervision. Compared with advanced international standards, China's GMP standards still have many disparities. In October 2010, with the World Health Organization GMP as the bottom line, referred to as the EU GMP standards, and considering the backward situation of domestic pharmaceutical companies, China revised its GMP again. Although there is still a particular gap between them and the GMP standards of developed countries, compared with China's old GMP version, the new version reflects significant improvements in technology and management. China stipulates that all pharmaceutical manufacturers must obtain the latest version of GMP certification before it allows production. Despite the improved standards, China's existing pharmaceutical companies present a situation of multiple, small, scattered and low levels, and their independent innovation capabilities are weak. The implementation of the new version of GMP is conducive to ensuring the quality of pharmaceutical production, to promoting the upgrading of China's pharmaceutical industry, and to enhancing the international competitiveness of Chinese pharmaceutical manufacturers.

## 3. Methods

The theoretical basis of this article is the theory of external signals. According to the external-signals theory, many regulators depend on information from external sources to make regulatory decisions. The information from external groups is important because it can reduce the uncertainties facing regulators and can lead to more defensible and perhaps more efficient decisions by them. Generally, regulators seek positive feedback from external groups. In contrast to positive feedback, negative feedback from external groups often causes trouble for regulators. Complaints about the decisions or behaviors of regulators may lead to government supervision, investigation or external attempts by interest groups to intervene in regulation activities, which are called troubles of regulators and often disrupts the regulators' operation. Increasing the efficiency of regulatory decisions may lead to more positive feedback from outside groups such as regulated firms, consumer groups, and politicians. The external-signals model provides a convenient framework for incorporating the role of such information into the process of regulator's decision making. This model suggests that regulatory decisions will be functions of the feedback from outside groups as well as of the information supplied by external sources to the regulator (32). In practice, regulatory measures taken by the government to protect consumers' health, life safety, living environment and public interests, include supervision and control of medicine's quality, efficacy, safety and so on. The special attributes of medicine determine that the main goal of government regulation is to ensure the quality and safety of medicine's production, prevent consumers from harming their health due to the use of drugs, and ensure their health and life safety. From the perspective of technical attributes of products and industries, government regulation of the pharmaceutical industry belongs to the category of social regulation, which is the regulation of the government on the quality of medicines and various activities accompanying them to establish specific standards, and to restrict and prohibit specific behaviors.

Due to market failures, countries around the world have generally strengthened government supervision in the medical and health fields. A crucial starting point is to ensure medicines' quality and safety. Noll (40) and Olson (41) proposed and developed the theory of external signals to explain which factors most concern regulatory agencies, and why regulators take the actions they do in response. For pharmaceutical manufacturers' supervision, the idea of external signals shows that a regulatory agency's actions are a function of a regulated company's characteristics, the features of risk management, the applicable regulatory policies, and the resources predetermined by the national macro-decision-making department. The goal is to maximize the regulatory agency's summation of the feedback signals received by the source. Following Luo (42), this paper adopts these four characteristics such as company's characteristics (size, ownership, financing, etc.); risk management characteristics (bank loan, annual quality review survey, multi-product collateral risk management, etc.); drug attribute characteristics (type of medicine, number of varieties produced throughout the year, etc.); the development characteristics demonstrated by the regulated company (proportion of R&D staff, expert help, etc.). The econometric model is (43):


(1) $$\begin{array}{l} GMP_{i}=\beta_{0}+\beta_{1}QY_{i}+\beta_{2}FX_{i}+\;\beta_{3}YP_{i}+\;\beta_{4}FZ_{i}+\mu_{i} \end{array}$$

The *GMP* on the left side of the above equation is the on-site inspection result of the drug enterprise, and *i* represents the inspected enterprise. The *QY* on the right side of the equation is the vector of corporate characteristic variables that affect the inspection results of pharmaceutical companies, such as the type of corporate ownership, the value of the company's fixed assets, the area of the purification zone, the operating time of the company, and the number of years the quality authorized person has entered the company. *FX* represents the variable vector of risk management characteristics, including whether the source of funds mainly relies on bank loans, whether to conduct annual quality review surveys, whether to establish multi-product collateral risk management, and whether to establish separate risk management procedures etc. *YP* represents the vector of drug characteristic variables, such as the type of drugs produced, the number of drug varieties produced throughout the year, the number of drug approval of the company, the efficiency of the company's capacity utilization, and laboratory maintenance costs, etc. *FZ* represents the development characteristic variable vector of the enterprise, including the proportion of enterprise R&D (research and development) personnel, personnel problems, whether to get expert help, the number of standard operating procedures, whether the enterprise expands and merges, and the enterprise quality system review cycle and other variables. μ_*i*_ is the random error term.

In the formula (1), on the one hand, the variable of the value of fixed assets represents the production capacity of the interviewed company, it has an impact on the company's GMP inspection results. On the other hand, the inspection results will also impact the company's value of fixed assets. As a result, there could be endogenous problems between the GMP inspection results and the company's fixed assets variables. The endogenous issue will most often lead to biased estimation. Building on the ideas of Qin et al. (44), in the survey area, we choose the average value of pharmaceutical companies' fixed assets as an instrumental variable for the value of fixed assets of pharmaceutical companies at the regional level. The reason for choosing this variable is that the average value of fixed assets at the regional level is closely related to the value of fixed assets of the interviewed company; however, it does not directly impact the results of the GMP inspection of the interviewed company. It is a more appropriate instrumental variable according to the rule to choose an instrumental variable. In fact, we use the value of a company's fixed assets that is 1 year behind to strengthen the instrumental variables and weaken the impact of the inspection results on the company's value of fixed assets.

GMP inspection results are discrete variables, and some studies use discrete choice models for quantitative estimation. However, Qin et al. (44) and Ferrer and Frijters (45) believe that the results obtained with the discrete model and the linear regression model estimated by the OLS method do not differ significantly, so this paper uses the linear probability model for its quantitative estimation. The other reason for using the linear probability model is that we also want to solve the endogenous problem between variables and need to use instrumental variables (IV) to do so. In contrast, discrete choice models such as the Ordered Logit model are nonlinear models and cannot directly use instrumental variables. Therefore, to calculate IV estimates, this article uses the two stage least square (2SLS) for quantitative testing. Simultaneously, the Multinomial Logit Model, the Ordered Logit and Ordered Probit model were used to test the robustness of the measurement results. In addition, we took the logarithm of the one-year lag of value of fixed assets to eliminate the non-normality of the data.

## 4. Results and discussion

### 4.1. Data sources and descriptive statistics

The data in this study comes from the actual inspection results of GMP in Anhui Province conducted by the State Drug Administration of China and face-to-face investigation of our research group. The investigation lasted for 3 years, covering the period from June 2017 to October 2019. The survey sample is selected by equidistant random sampling method. There are 380 pharmaceutical companies in Anhui Province, of which 294 are selected to be surveyed. The proportion of surveyed companies in all pharmaceutical companies in Anhui Province is 77%. The survey data consists of two parts. The first part is the actual result of the routine GMP normative inspection conducted by the inspectors of Anhui Provincial Drug Administration. As mentioned before, the inspectors of the Drug Administration are professionals in this field. They can only obtain the inspection qualification after passing the strict qualification examination. The second part is for the inspected companies. When inspectors of the Drug Administration carry out on-site inspection of the company, the investigators of the research group enter the company to conduct a questionnaire interview on the quality of the company's drug production and management, focusing on the company's characteristics and risk quality management. Investigators of the research group are allowed to participate in formal research only after formal training and passing examinations. After the investigation, the two parts of data are combined to build a complete data set which contains the GMP inspection results and condition of business for each company, forming the basic data for this study.

FDA inspectors conduct their on-site inspections following the Good Manufacture Practice of Medical Products (revised in 2010) promulgated by China's Ministry of Health. The GMP specification covers a total of 829 indicators. According to the seriousness of defects, each indicator's inspection results are divided into four categories: standard, minor defects, major defects, and severe defects, labeled 0, 1, 2 and 3, respectively. If an inspector uncovers a severe defect, the company is deemed to have failed the inspection. Consequently, when its GMP certificate expires, a company cannot continue to produce drugs, and offenders will be punished as drug counterfeiters. In severe cases, they can be held criminally responsible. Typically, after a GMP certificate expires, it will automatically be renewed for another 5 years if its other results are satisfactory, allowing the company to continue production.

Additionally, the result of a severe defect (failed) includes two situations: first, if any enterprise under inspection has one or more 3 ratings (i.e., severe defects) in all 829 indicators; second, if there are four and above 2 ratings (i.e., major defects). We have organized the results of the GMP inspection of 829 indicators into a total indicator that conveys the overall rating: if only 0 and no other values appear, it means normal and a value of 0 is assigned; if at least one 1 appears but no 2 or 3, then this indicates a minor defect and a value of 1 is assigned; if there are three or less 2s but no 3, it means a major defect and a value of 2; if there is at least one 3, or four or more 2s, it means a severe defect (not a pass) and the value 3 is assigned. The final inspection result is a combination of the National Medical Products Administration's results and the dependent variable used in this article. None of the companies surveyed for the paper received a final result of 0; the numbers of companies identified as having minor defects, major defects, and severe defects (failed) are 85, 164, and 45, respectively. It can be seen that the proportion of severe defects (not passed) is 15.31%.

Table 1 provided a comparison of GMP inspection results of enterprises with different ownership types. Among the 294 enterprises in the sample, the numbers of state-owned enterprises, private enterprises, and foreign-owned enterprises were 14, 265 and 15, respectively. Among the variables of enterprise ownership types, the various enterprises' sample proportions are consistent with the country's overall proportion. In this study, none of the foreign companies had severe defects (failed) in their inspection results, indicating that the foreign companies maintain relatively high GMP standards. The proportion of private enterprises with severe defects (failed) in the inspection results was 15.85%, but the proportion of state-owned enterprises with severe defects (failed) was as high as 21.43%. Compared with foreign-owned enterprises, both state-owned and private enterprises have big gaps in their production standardization. Table 2 compares GMP inspection results of the companies whether bank loan is their funding source. For companies that use bank loans as their primary source of funding, poor GMP inspection results can have severe consequences.

**Table 1:** Comparison of GMP inspection results of enterprises with different ownership types.

**GMP inspection results**	**Minor defects (1)**	**Major defects (2)**	**Severe defects (i.e., failed) (3)**	**Total defects (4)**	**Proportion of severe defects (3)/(4)**
**Type of enterprise**					
**State-owned enterprises**					
Amount (unit)	4	7	3	14	21.43%
Proportion (%)	4.71	4.27	6.67	4.76	
**Private enterprises**					
Amount (unit)	73	150	42	265	15.85%
Proportion (%)	85.88	91.46	93.33	90.14	
**Foreign-owned enterprises**					
Amount (unit)	8	7	0	15	0%
Proportion (%)	9.41	4.27	0	5.1	
**Total**					
Amount (unit)	85	164	45	294	15.31%
Proportion (%)	100	100	100	100	

F-test is significant at the level of 10% (*P* = 0.0780).

**Table 2:** Comparison of GMP inspection results of the main enterprise whether the source of funds depends on bank loans.

**GMP inspection results**	**Minor defects (1)**	**Major defects (2)**	**Severe defects (i.e., failed) (3)**	**Total defects (4)**
**Funding source**				
**Not based on bank loans**				
Amount (unit)	55	86	19	160
Proportion (%)	64.71	52.44	42.22	54.42
**Mainly based on bank loans**				
Amount (unit)	30	78	26	134
Proportion (%)	35.29	47.56	57.78	45.58
**Total**				
Amount (unit)	85	164	45	294
Proportion (%)	100	100	100	100

F-test is significant at the level of 5% (*P* = 0.0370).

**Figure 1:**
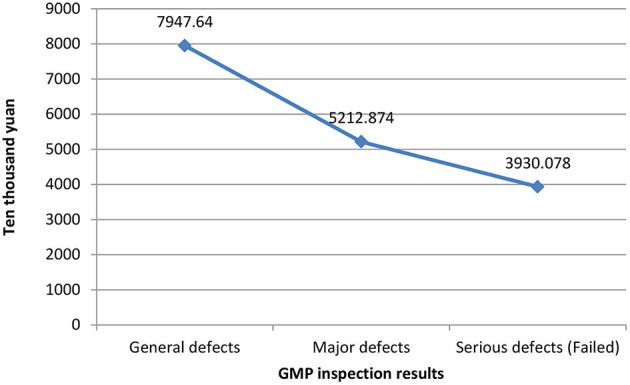
Fixed assets values and GMP inspection results.

**Figure 2:**
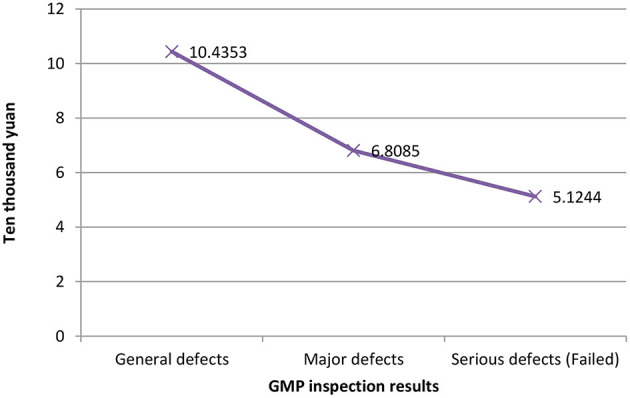
Years of quality authorized staff in the company and GMP inspection results.

Figure 1 shows the relationship between different companies' fixed assets values and their GMP inspection results. The vertical axis represents the value of fixed assets, and the horizontal axis represents the GMP inspection results. It can be seen that the more the value of fixed assets, the better the results of the company's GMP inspection, vice versa. The relationship between the years of quality authorized staff and GMP inspection results is shown in Figure 2. The vertical axis is the number of years of quality authorized staff entering the company, and the horizontal axis is the GMP inspection results. The figure clearly shows that the longer the quality authorized staff has entered the company, the better the company's GMP standard result. However, the less the quality authorized person has entered the company, the more severe the GMP inspection results of the company will be (failed). These results preliminarily show that the value of fixed assets and the variable of the number of years of quality authorized staff have a negative correlation with the results of GMP inspections.

The value of the company's fixed assets ranges between 1.344 billion RMB Yuan (~1 USD = 6.75 RMB Yuan, year of 2020) and only 5 million RMB Yuan. This wide span shows that the scale of Chinese enterprises is quite different from western counterparts, and there will be big gaps in production capacity and production levels. The area of the purification area is 7,596 square meters for the largest enterprise, and there is no purification area for the smallest. The company's operation has been running for an average of 14 years, with the shortest term being 3 years. The operation time of an enterprise represents the production of its experience. The longer the operation history, the richer the production experience of the enterprise and the more helpful it is to standardize production. The longest quality authorized person has served for 36 years, and the shortest for only 1 year. For quality authorized personnel, 25.85% have a college degree or below, 66.33% have a bachelor degree, and only 7.82% have a Master degree or above. Quality authorized persons have special responsibilities in the GMP standard production process of drugs, and their education level needs to be improved. As much as 45.58% of enterprises, the source of funds in the process of GMP comprehensive transformation and implementation mainly relies on bank loans. It shows that the strength of the enterprise is weak, and the ability of self-financing is low. The definitions and descriptive statistics of other variables are shown in Table 3.

**Table 3:** The definition of variables and descriptive statistics in this study.

**Variables**	**Definition**	**Mean**	**Standard deviation**	**Minimum**	**Maximum**
GMP inspection results	The GMP inspection results that are conducted by the FDA, the minor defect is 1, the major defect is 2, and the severe defect (failed) is 3.	1.8639	0.6520	1	3
**Type of enterprise ownership**					
State-owned enterprises	State-owned enterprise is 1, otherwise is 0	0.0476	0.2133	0	1
Private enterprises	Private enterprise is 1, otherwise is 0	0.9014	0.2987	0	1
Foreign enterprises	Foreign enterprise is 1, otherwise is 0	0.0510	0.2204	0	1
Fixed assets values	Value of the company's fixed assets (10,000 yuan)	5,857.6010	13,032.8700	500	134,449
Area of purification zone	No more than 100,000 bacteria/square meter area	155.7564	649.192	0	7,596
Enterprise operating time	Number of years since the establishment of the company (years)	14.0680	17.0409	3	214
Years of quality authorized staff in the company	Number of years the quality authorized staff has worked in the company (years)	7.6139	7.4411	1	36
**Education of quality authorized staff**					
College degree and below	If the enterprise quality authorized staff obtained a college degree or below, the degree is 1, otherwise is 0	0.2585	0.4386	0	1
Bachelor degree	If the enterprise quality authorized staff obtained a bachelor degree, the degree is 1, otherwise is 0	0.6633	0.4734	0	1
Master degree and above	If the enterprise quality authorized staff obtained a master degree or above, the degree is 1, otherwise is 0	0.0782	0.2690	0	1
Bank loan	The source of corporate funds mainly relies on bank loans as 1, otherwise is 0	0.4558	0.4989	0	1
Annual quality review survey	The annual quality review survey for drug batches that do not meet the quality standards is 1, otherwise is 0	0.7211	0.4492	0	1
Multi-product collateral risk management	Established a multi-product collateral risk management procedure for 1, otherwise is 0	0.5510	0.4982	0	1
Individual risk management procedures	Established a separate risk management procedure for 1, otherwise is 0	0.9626	0.1901	0	1
**Type of medicine**					
Medical oxygen	The production of medicines belongs to medical oxygen is 1, otherwise is 0	0.0442	0.2059	0	1
Chinese medicine decoction pieces	The production of medicines belong to Chinese medicine decoction pieces is 1, otherwise is 0	0.5272	0.5001	0	1
Preparation drugs	The production of medicines belong to Preparation drugs is 1, otherwise is 0	0.3605	0.4810	0	1
Sterile drugs	The production of medicines belong to Sterile drugs decoction pieces is 1, otherwise is 0	0.0680	0.2522	0	1
Number of varieties produced throughout the year	The number of types of drugs produced by the enterprise throughout the year (pieces)	149.2755	197.8091	1	871
Number of approved document numbers	Number of drug approval document numbers owned by the enterprise (pieces)	20.5102	45.5893	1	396
Capacity utilization	Annual capacity utilization rate (%)	61.3655	24.5813	10	100
Laboratory maintenance costs	Annual maintenance cost of the laboratory (10,000 yuan)	32.4241	95.6821	6	1,224
Proportion of RandD staff	The proportion of RandD staff to the total number of employees (%)	4.4796	6.8108	0	37
Staff issues	In the process of implementing GMP, the company considers the seriousness of the operating personnel's problems. 1, 2, 3, 4, and 5 respectively indicate: no, very little, general, more, and very much	3.0102	1.0368	1	5
Expert help	In the process of implementing GMP, the extent to which the company believes that it has received expert help, 1, 2, 3, 4, and 5 respectively indicate: no, very little, general, more, and very much	2.8299	1.1108	1	5
Number of standard operating procedures	Number of standard operating procedures established by the enterprise (unit)	578.6905	756.9782	6	7,798
Expand merger	The enterprise development strategy is to expand the merger to 1, or not to 0	0.1190	0.3244	0	1
Quality system review cycle	The quality system review cycle is 1 over half a year, and 0 within half a year	0.1565	0.3639	0	1

### 4.2. Main model results

Table 4 shows the regression results from the 2SLS model for examining the influencing factors of the corporate GMP inspection results. This is the main model result in this paper. At the same time, as shown under Model (1), this table also lists the measurement results of the OLS model. The coefficient values of Model (2) are all greater than those of the corresponding variables of Model (1), indicating that if the endogenous problem is not solved, the impact of variables such as enterprise characteristics and risk management characteristics on the results of GMP inspections will be underestimated. Among the enterprise characteristic variables of Model (2), the results of enterprise ownership type variables show that compared with state-owned enterprises, private and foreign enterprises have relatively better inspection results. In fact, the inspections of foreign pharmaceutical companies revealed no severe defects (fails). These companies are generally branches of relatively strong international pharmaceutical enterprises and have passed the stringent GMP certification processes in places such as Europe and the United States. Their adherence to production process guidelines and internal control indicators are relatively strict, and the degree of standardization company to company is high. Comparing national GMP standards, China's are inherently lower than those of major economies such as the United States, and its state-owned enterprises and private enterprises still lack GMP hardware and software investment. Overall, China companies' production concepts, quality awareness, and management techniques are in urgent need of improvement.

**Table 4:** 2SLS method regression results of factors affecting the results of GMP inspection of pharmaceutical companies.

**Variables**	**OLS model**			**2SLS model**		
	**Model (1)**			**Model (2)**		
	**Coefficients**	**SD**	***T*** **-value**	**Coefficients**	**SD**	***T*** **-value**
**Characteristics of enterprises**						
**Type of enterprise ownership (reference group: State-owned enterprises)**						
Private enterprises	−0.3903^**^	0.1932	−2.0200	−0.6157^***^	0.2487	−2.4800
Foreign enterprises	−0.5458^**^	0.2341	−2.3300	−0.6422^***^	0.2553	−2.5200
Total fixed assets values	−0.0555	0.0345	−1.6100	−0.2533^**^	0.1292	−1.9600
Area of purification values	−0.0001	0.0001	−1.2600	−0.0001	0.0001	−0.9800
Enterprise operating time	−0.0030	0.0026	−1.1600	−0.0019	0.0028	−0.6600
Years of quality authorized staff work in the company	−0.0143^**^	0.0063	−2.2900	−0.0129^**^	0.0067	−1.9300
**Quality authorized staff's degree (reference group: College degree and blow)**						
Bachelor	0.2005^**^	0.0863	2.3200	0.2593^***^	0.0986	2.6300
Master and above	−0.0465	0.1524	−0.3100	0.0598	0.1747	0.3400
**Characteristics of risk management**						
Bank loan	0.1843^**^	0.0749	2.4600	0.2335^***^	0.0852	2.7400
Annual quality review survey	−0.2759^***^	0.0855	−3.2300	−0.2627^***^	0.0909	−2.8900
Multi-product collateral risk management	−0.1459^*^	0.0828	−1.7600	−0.0875	0.0951	−0.9200
Individual risk management procedures	0.4419^**^	0.1967	2.2500	0.4852^**^	0.2102	2.3100
**Characteristics of drugs**						
**Type of medicine (reference group: Medical oxygen)**						
Chinese medicine decoction pieces	−0.0524	0.2038	−0.2600	−0.1435	0.2234	−0.6400
Preparation drugs	−0.0002	0.2015	0.0000	0.0107	0.2137	0.0500
Sterile drugs	0.1759	0.2553	0.6900	0.1824	0.2706	0.6700
Number of varieties produced throughout the year	0.0003	0.0002	1.1500	0.0004	0.0003	1.4800
Number of approved document numbers	0.0005	0.0010	0.4600	0.0016	0.0013	1.2400
Capacity utilization	0.0019	0.0015	1.2900	0.0015	0.0016	0.9600
Laboratory maintenance costs	0.0206	0.0419	0.4900	0.0204	0.0444	0.4600
**Characteristics of development**						
Proportion of RandD staff	0.0025	0.0058	0.4400	0.0001	0.0064	0.0100
Staff issues	0.0292	0.0368	0.7900	0.0388	0.0394	0.9800
Expert help	−0.0199	0.0343	−0.5800	−0.0200	0.0363	−0.5500
Number of standard operating procedures	0.0000	0.0001	0.5200	0.0000	0.0001	0.6600
Expand merger	−0.0395	0.1151	−0.3400	−0.0849	0.1253	−0.6800
Quality system review cycle	0.1447	0.0994	1.4600	0.1516	0.1054	1.4400
Constant term	2.1989^***^	0.4454	4.9400	3.7765^***^	1.0953	3.4500
Adj R-squared	0.1559			0.0521		
*N*	294			294		

^***^, ^**^, and ^*^ indicate significant at the statistical level of 1, 5, and 10%, respectively. SD refers to standard deviation.

The results of the value of fixed assets show that when the value of a company's fixed assets is higher, the probability that the company's GMP inspection result will be seriously defective (i.e., fail) is significantly reduced. From another perspective, the lower the value of the company, the higher the probability that inspection will reveal severe defects (failures). The value of fixed assets represents the scale of the enterprise to a certain extent, with a high value of fixed assets invariably indicating a large-scale enterprise. Larger companies tend to have wide-ranging social influence, and severe product-quality problems will greatly damage the company's social reputation, reduce its image in the eyes of consumers, and interfere with market success. If a company has problems, the company will suffer greater losses, and the cost and price paid for regaining reputation and the market are also greater. It can be seen that companies with higher fixed assets have higher inherent requirements, pressures and motivations for standardized production. Therefore, strengthening the GMP supervision of enterprises with low fixed assets is one of the key work directions of the FDA in the future.

The estimation results of years of quality authorized staff in the company show that the longer time of the staff works in the company can significantly promote the level of GMP inspection result and reduce the risk of company's inspection results as severe defects (failed). Many years of practical experience in developed countries have shown that the quality authorized person system is an effective drug quality management system, which helps companies to ensure drug quality and fulfill social responsibilities. The main responsibilities of the quality authorized person include not only implementing the laws and regulations on drug quality management, organizing and regulating the company's drug production quality work; but also organizing, establishing and improving the production quality management system of the pharmaceutical company, and monitoring it to ensure effective operation. For the selection of key material suppliers, the production equipment, and key staff in production, quality, materials, equipment and engineering departments, the quality authorized staff has the right to decide and veto. The quality authorized staff is specially assigned and required to perform the function of product release. Once the product is released, it will directly face the patients, and the quality of the product will affect the life safety of the patient. In fact, the product release function is the core function of GMP, which runs through the entire GMP system. In addition to the solid professional knowledge of the quality authorized staff, the understanding of the company's factory conditions, the grasp of the production skills and specialties of the company's employees, and the knowledge of the company's production facilities are all long-term requirements for these quality authorized staff.

Among the risk management characteristic variables, the regression results of the bank loan variables show that if a company mainly relies on bank loans for implementing GMP, the GMP inspection has a higher probability of identifying severe defects (failure). This trend can be explained by the nature of the modern pharmaceutical production industry, which is both technology-intensive and capital-intensive (46), meaning that producers face huge capital outlays when forced to modernize their production technologies. Before implementing GMP certification standards, there were big differences in the production methods between different pharmaceutical manufacturers in China, and there were also certain differences in product quality. Moreover, a big gap existed between Chinese production lines and the pharmaceutical production standards of developed countries. Chinese pharmaceutical manufacturers needed to renovate their original plants and equipment and other software and hardware following the GMP regulations; moreover, subsequent production facilities had to continue to comply with GMP's standards. Regardless of the original enterprise's comprehensive GMP transformation, or the maintenance of GMP production specifications after the transformation, huge investment is still required. If the enterprise can barely meet the needs of GMP transformation and development with self-raised funds, it will be burdened with greater repayment pressure from banks and other investors it borrowed from to make the transformation. This burden will increase the enterprise's operating costs and is not conducive to market competition. Generally speaking, after passing the GMP certification, the company's unit cost will have increased by nearly 20% on average due to equipment renewal, plant depreciation, and increased management costs. Companies that rely mainly on bank loans to implement GMP tend to lack self-owned funds, are small and relatively weak with regards to resources such as staff and financial backing, and usually have poor production capacity.

The results of other variables are also worthy of attention. Examining the time an enterprise has been in operation often shows that the longer a company has been established, the lower the probability of severe defects being found during inspection. However, this result is not statistically significant. Measuring the variables of a company's annual quality internal review survey usually indicates that if the company finds drug batches that do not meet quality standards, it will increase its production diligence. Consequently, the probability of it failing a later GMP inspection is significantly reduced. The purpose of the annual quality review is to ensure the stability and reliability of the production process, and to ensure that the transition of raw materials into finished pharmaceutical products meets the requirements of the current GMP, and that the quality of drugs meets the predetermined standards. The establishment of a multi-product co-line risk management system has improved the level of GMP production standards. Companies have noted that previous lack of quality awareness in employees led to the occurrence of GMP production irregularities in the past. The guidance and help of industry experts have since improved the standardization of GMP production.

### 4.3. Further analysis

Table 5 shows the regression results of the Multinomial Logit model of the factors such as the enterprise characteristics and risk management, affecting the GMP inspection results both the major defects and the severe defects, i.e., failed. First, the smaller the value of fixed assets, the higher the probability that the inspection result will be a severe defect. Second, the longer the quality authorized staff has been in the company, the more likely it is that the company's inspection result is a severe defect. Third, companies that mainly rely on bank loans will have worse inspection results. Lastly, if the annual quality review survey is not conducted, the probability that the company's inspection results fail will be significantly increased. From the perspective of drug types, compared with the medical oxygen category, the production of Chinese medicine decoction pieces, preparation drugs, and sterile drugs, the inspection results of these three types of drugs are severe defects, that is, the probability of failing is higher.

**Table 5:** Multinomial Logit model regression results of factors affecting GMP inspection results of pharmaceutical companies.

**Variables**	**GMP inspection result is the major defect**			**GMP inspection result is the severe defect**		
	**Model (1)**			**Model (2)**		
	**Coefficients**	**SD**	**Z-value**	**Coefficients**	**SD**	**Z-value**
**Type of enterprise ownership (reference group: State-owned enterprises)**						
Private enterprises	−1.5776^**^	0.8224	−1.9200	−2.0432^*^	1.0950	−1.8700
Foreign enterprises	−1.3523	0.9280	−1.4600	−17.0599	1521.4800	−0.0100
Fixed assets values	−0.2889^**^	0.1494	−1.9300	−0.3727^*^	0.2232	−1.6700
Years of quality authorized staff work in the company	−0.0110	0.0271	−0.4100	−0.1066^**^	0.0469	−2.2700
Bank loan	0.7894^**^	0.3413	2.3100	1.1656^***^	0.4666	2.5000
Pseudo *R*^2^	0.1915					
*N*	294			294		

(1) The result of the GMP inspection in the above table is that the minor defect is used as the reference group; (2) ^***^, ^**^ and ^*^ indicate significant at the statistical level of 1, 5, and 10%, respectively; (3) The other controlled variables same as those in Table 4.

### 4.4. Robustness test

Table 6 reports the econometric regression results of the ordered dependent model. Its purpose is to test the stability of the regression results of each variable. On the one hand, the results of the Ordered Logit model and the Ordered Probit model are basically similar; on the other hand, compared to the estimation results of the 2SLS model (Table 4), the econometric regression results of the ordinal dependent variable model (Table 6), not only, for each variable, the signs of the coefficient are basically the same, but also the significance of the estimated values of the corresponding variable coefficients are the same. This indicates that the econometric regression results in this paper are relatively robust. These results also show that if these corporate characteristics and quality risk management characteristics that have a greater influence on the results of GMP inspections are used as the focus, strengthening the supervision of pharmaceutical manufacturers will help improve the degree of GMP regulations of pharmaceutical manufacturers. It also helps to ensure the production quality of medicines.

**Table 6:** The regression results of the ordinal dependent variable model of the influencing factors of the GMP inspection results of pharmaceutical companies.

**Variables**	**Ordered Logit model**			**Ordered Probit model**		
	**Model (1)**			**Model (2)**		
	**Coefficients**	**SD**	***Z*** **-value**	**Coefficients**	**SD**	***Z*** **-value**
**Type of enterprise ownership (reference group: State-owned enterprises)**						
Private enterprises	−1.5325^**^	0.6975	−2.2000	−0.8580^**^	0.3827	−2.2400
Foreign enterprises	−2.0196^**^	0.8295	−2.4300	−1.1964^***^	0.4741	−2.5200
Fixed assets values	−0.1963^*^	0.1178	−1.6700	−0.1151^*^	0.0679	−1.6900
Years of quality authorized staff work in the company	−0.0531^**^	0.0222	−2.3900	−0.0319^***^	0.0129	−2.4800
Bank loan	0.6402^***^	0.2594	2.4700	0.3787^***^	0.1484	2.5500
Pseudo *R*^2^	0.1346			0.1351		
*N*	294			294		

(1) ^***^, ^**^, and ^*^ indicate significant at the statistical level of 1, 5, and 10%, respectively; (2) The other controlled variables same as those in Table 5.

## 5. Conclusion and discussion

Based on the on-site inspection and survey data of GMP of pharmaceutical companies in Anhui Province of China, by the National Medical Products Administration, this paper empirically tests the impact of corporate characteristics and risk management on the GMP regulations. The empirical research in this article provides an essential practical basis for academic research and policy maker. It also provides a scientific basis for any country to improve the targeted and effective supervision of pharmaceutical companies. Four research results are found. First, both foreign-owned and private enterprises have a higher GMP result than state-owned enterprises. Second, a company's GMP inspection results are worse when its source of funds is more dependent on bank loans. Third, for companies with less fixed assets, their GMP inspection results are worse. Fourth, the shorter the quality authorized employee has been with a company, the worse that company's GMP inspection results. To improve the pertinence of pharmaceutical manufacturers' supervision, strengthen the GMP standardization of pharmaceutical production, and ensure the production quality of pharmaceuticals, the above aspects should be taken as the entry point for supervision.

Contrasting with foreign-owned and private enterprises, state-owned enterprises are both market entities and administrative entities privileged, and they can obtain additional resources. State-owned enterprises began overlooking and disregarding quality responsibility, caused by many enterprises having ambiguous budget constraints and setting excessive non-business objectives, plus the non-marketization of incentive mechanisms caused by the government. The root cause is these state-owned enterprises are protected by government policy and are given resource monopoly, which is causing some serious consequences. State-owned enterprises have become the priority development targets at all levels of government with absolute financial support, exclusive policy resources, strong economic strength, and funds that are ultimately “exhausted” by the finances. Some have even become “shadow banks”, where they could obtain loans at extremely low rates due to their special privileges, and then lend out those loans at higher rates to private enterprises.

One of the important driving forces of China's economic growth miracle since the period of Reform and Opening-up is that private enterprises have obtained a wider space for development. The people's enthusiasm for innovation and entrepreneurship has been released explosively; private enterprises have served the entire society at a much higher efficiency than state-owned enterprises. It also creates wealth for capital holders. In the market economy environment, the competition of various enterprises is a comprehensive competition of product quality and efficiency. Ensuring quality, adhering to integrity, fair trading, and respect for market rules is the long-term survival of all enterprises, including state-owned enterprises. All enterprises registered in China must establish equal status in law and treat them equally in terms of policies to provide a market basis for fair competition among all enterprises. Simultaneously, we must adhere to supply-side structural reforms' mainline and accelerate market-oriented reforms in the financial sector. China's financial institutions are not yet market entities in the true sense. At present, a considerable number of financial institutions in China have inadequate restraint and incentive mechanisms. Illegal lending and non-market-based lending operations occur from time to time, which increases potential financial risks and is not conducive to the development of the real economy, including pharmaceutical companies. And product quality control. For corporate loans, it should be resolved through market means such as market financing and personal lending. Further, it is necessary to strengthen the legislative work in the financial field, improve the financial market's level of supervision, promote the commercialization of banks, and establish a new market-oriented bank-enterprise relationship.

Although this paper is a research on how to strengthen the supervision of pharmaceutical production companies from the perspective of drug supervisors, we still need to consider how to protect the quality of drug production from government departments and enterprises. From a government department, local protectionism is subject to local protectionism to strengthen the concentration of drug production enterprises. China's drug production enterprises have more than 7,000, and only more than 100 pharmaceutical companies such as USA and Japan and other pharmaceutical powers. The top three drug production enterprises, China's concentration is 6.16%, while the United States reached 32%; for the top 10 drug production enterprises, China's concentration is 14.26%, and the United States is as high as 63%, Japan also reached 52% (47). With lower concentration, China's pharmaceutical production enterprises have poor economic benefits, and the homogenization of products is serious. For example, the number of NOFCS drug production enterprises is as high as more than 800. More than 80% of pharmaceutical companies in China are small and medium-sized enterprises, the production equipment is relatively small, and the production equipment is older, and the problem with the management level is very prominent (1). Affected by the economic concept of local development, local protection, government supervision is not in place, leading to the quality awareness of the company, thus affecting the production standards and the quality of the drug, and will ultimately endanger the lives of the people. For companies with weak funds, we must dare to face their own market position, we must work with large enterprises, or become a subsidiary of large enterprises, to improve their production level. However, these companies cannot blindly rely on compliant or non-compliant means to obtain external funds to maintain their own survival. State-owned enterprises and private enterprises should also do everything possible to learn GMP standard production experience to improve their production levels and meet the requirements of GMP.

Although this paper has created an exclusive empirical study in GMP drug production supervision, there are still some shortcomings. First, the number of sample companies has to be increased. In the existing literature study in this field, the sample amount used herein is relatively, but the representation of the sample can be further enhanced if the number of observed values of the existing samples can be largely expanded. Second, the sample uses cross-sectional data and cannot eliminate the influencing factors that do not change over time. As mentioned above, the non-openness of the GMP examination results, the professional data collection, and the huge cost of the on-site inspection, are the biggest obstacles to data acquisition and are also fundamental reasons that are difficult to have a breakthrough in this research area. In the future, we should strengthen cooperation with relevant departments, carry out continuous long-term planning, and collect data on a larger scale and for a longer period to further study this challenging area.

## Data availability statement

The original contributions presented in the study are included in the article/supplementary material, further inquiries can be directed to the corresponding authors.

## Author contributions

Conceptualization: HC and LQ. Methodology: LQ and YS. Software: MQ and LQ. Writing: CJ and MQ. Formal analysis: HC, CJ, YS, and JL. All authors contributed to the article and approved the submitted version.
